# Retroperitoneoscopic single site renal biopsy surgery: right indications for the right technique

**DOI:** 10.1186/1471-2490-14-80

**Published:** 2014-10-13

**Authors:** Salvatore Micali, Alessio Zordani, Riccardo Galli, Eugenio Martorana, Micaela Piccoli, Gianni Cappelli, Giampaolo Bianchi

**Affiliations:** Policlinico di Modena, Department of Urology, University of Modena and Reggio Emilia, Via del Pozzo 71, Modena, 41124 Italy; Department of Nephrology, University of Modena and Reggio Emilia, Modena, Italy; Department of General Surgery, Baggiovara Hospital, Modena, Italy

**Keywords:** Retroperitoneoscopy, Single-site surgery, LESS, Renal biopsy

## Abstract

**Bacground:**

Laparoendoscopic single-site surgery (LESS) has been developed in an attempt to further reduce the morbidity and scarring associated with laparoscopic surgery. In patients in whom there are indications to perform a laparoscopic renal biopsy, LESS surgery is a valid alternative to mini invasive surgery and is becoming more common. We report our experience on 14 renal biopsy procedures performed in a retroperitoneal LESS.

**Methods:**

LESS renal biopsy was performed in 14 patients 18 to 80 years old (mean age 58.3 years) during a 36 month period. All procedures were performed by a single operator. The patient was in a standard flank position. The procedure was performed using a 2.5 cm, single incision via a retroperitoneal access at the Petit’s triangle. A 5 mm biopsy forceps was used to collect the specimen under direct vision, and haemostasis was obtained with an Argon beam probe and the application of oxidized regenerated cellulose gauze.

**Results:**

Biopsy was performed successfully in all cases. Mean operative time was 52.64 min, blood loss was minimal, and the hospital stay ranged from 12 to 24 hours. None of the patients required narcotics or additional analgesia in the postoperative period. No postoperative complications occurred.

**Conclusions:**

The LESS technique is safe, reliable (100% success), easy to learn, and offers subjective cosmetic benefits to the patient. Minimal hospitalization requirement following retroperitoneal LESS biopsy is an additional timely advantage over laparoscopic renal biopsy. We think that with the right indications (marked obesity, failure of previous percutaneous biopsy attempts, a solitary kidney and coagulopathy) LESS renal biopsy is a good alternative to laparoscopy. Our next step will be a randomized prospective study of LESS compared with laparoscopy for renal biopsy to support our findings.

## Background

Histological evaluation of renal parenchyma is often essential in cases of several renal diseases with unexplained azotemia, proteinuria, hematuria, or systemic disease. Pathological diagnosis often provides useful information in determining the prognosis and guiding the treatment. Ultrasound-guided renal biopsy represents an essential step in the study of renal diseases [1].

The last few decades have transformed the renal biopsy into a safe technique that plays a central role in the nephrological diagnostic approach [2].

With percutaneous renal biopsy, as many as 5 to 20% of cases yield inadequate tissue for histopathology diagnosis. Moreover, percutaneous kidney biopsy is not without risk. Over complications occurring in up to 13% of the cases, and 6 to 7% of complications were considered major, needing for an intervention such as transfusion of blood product or invasive procedure (radiographic or surgical) [3].

Difficulty in localized and inaccurate biopsies may be taken when patients are extremely obese, uncooperative, or have an ectopic kidney or abnormal body habitus [4].

Gimenez et al. describes the first retroperitoneal laparoscopic renal biopsy technique in 32 patients who previously failed ultrasound-guided biopsy and in whom the approach was contraindicated [5]. Relative indications for the laparoscopic approach include marked obesity [6], failure of previous percutaneous biopsy attempts, a solitary kidney, a coagulopathy, Jehovah’s Witness faith, and in pediatric patients [7]. Laparoscopic renal biopsy is preferred in which the retroperitoneal laparoscopic approach is able to obtain sufficient renal tissue, with minimal bleeding complications and a minimally invasive approach.

With advances in endoscopic instrumentation and the development of laparoscopic techniques, the minimally invasive renal biopsy is safety and preferable [8]. Retroperitoneal laparoendoscopic single-site surgery (LESS) has been developed in an attempt to further reduce the morbidity and scarring associated with laparoscopic surgery. Early clinical series have demonstrated the feasibility of a broad range of retroperitoneal LESS urologic procedures [9]. We present our preliminary experience with retroperitoneal LESS in a series of 14 subjects who required renal biopsy.

## Methods

### Patient demographics

Between January 2011 and December 2013, 14 patients (11 male and 4 female) between 18 to 80 years old (mean age 58.8 years) were referred to our division for retroperitoneal LESS for renal biopsy. All these patients had absolute contraindications for a percutaneous renal biopsy*.*

All patients had abnormal proteinuria and or renal insufficiency, defined by a serum creatinine of >1.4 mg/dL (14 cases) and were referred for a LESS renal biopsy. All patients had undergone a complete evaluation by a nephrologist beforehand. Proteinuria ranged between 75 to 721 mg/dL; average serum creatinine was 2.15 mg/dL (range: 0.98 to 4.98). Three patients had morbid obesity; mean body mass index was 55 kg/m^2^ (range: 51.3 to 60.5). Of the entire group, three patient were ASA (American Society of Anesthesia) class 1, eight were ASA class 2, and three were ASA class 3 (morbid obesity). The indications for pursuing a laparoscopic approach are listed in Table 1.

**Table 1:** **Patient indications for performing LESS renal biopsy, surgical data and histopatological diagnosis based on retroperiotneoscopic LESS renal biopsy**

Age	Disease	Technique	OT	Blood loss	ANP	HS	Histopatological diagnosis
55	Extremely obese	Hybrid single trocar	85	< 50 cc	No	1 day	GN membranoproliferative
40	Coagulopathy	Hybrid single trocar	105	< 50 cc	No	1 day	Membranous glomerulonephritis
66	Extremely obese	Hybrid single trocar	75	< 50 cc	No	1 day	IgA nephropathy
62	Solitary kidney	Pure single trocar	90	< 50 cc	No	1 day	GN membranoproliferative
58	Solitary kidney	SILS Port/ Bariatric Laparoscope	38	< 50 cc	No	1 day	FSGS
72	Uncontrolled ipertension	SILS Port/ Bariatric Laparoscope	45	< 50 cc	No	1 day	Membranous glomerulonephritis
79	Uncontrolled ipertension	SILS Access Port/ Endo EyE	35	< 50 cc	No	1 day	GN membranoproliferative
77	Uncontrolled ipertension	SILS Access Port/ Endo EyE	27	< 50 cc	No	1 day	AL amyloidosis
65	Extremely obese	SILS Access Port/ Endo EyE	24	< 50 cc	No	1 day	FSGS
59	Solitary kidney	SILS Access Port/ Endo EyE	27	< 50 cc	No	1 day	Membranous glomerulonephritis
80	Solitary kidney	SILS Access Port/ Endo EyE	45	< 50 cc	No	1 day	FSGS
22	Solitary kidney	SILS Access Port/ Endo EyE	39	< 50 cc	No	1 day	GN membranoproliferative
18	Solitary kidney	SILS Access Port/ Endo EyE	42	< 50 cc	No	1 day	GN membranoproliferative
70	Solitary kidney	SILS Access Port/ Endo EyE	60	< 50 cc	No	1 day	AL amyloidosis

OT: Operative time in minutes; ANP: Analgesia Post Surgery; HS Hospital Stay.

Patients were fully informed about the risks, benefits, alternatives, personnel and the novelty of Retroperitoneal LESS Renal Biopsy. Before surgery, we ensured that each patient had provided consent for this procedure. They were informed that retroperitoneal access via Petit’s Triangle would be secured as a matter of routine in each case and that, at the surgeon’s discretion, the procedure would be converted to standard laparoscopic surgery on failure to progress. Each patient was informed that such elective conversion to standard laparoscopy was not a complication but rather surgical prudence because of the novelty of this procedure. All data were collected retrospectively in excel file without any patient identifying information. The techniques used were all part of routine care at Department of Urology University of Modena and Reggio Emilia, and since each patient signed a general consent to the processing of personal data, formal ethical approval from our institutional review board was not necessary.

### Operative technique

**Figure 1:**
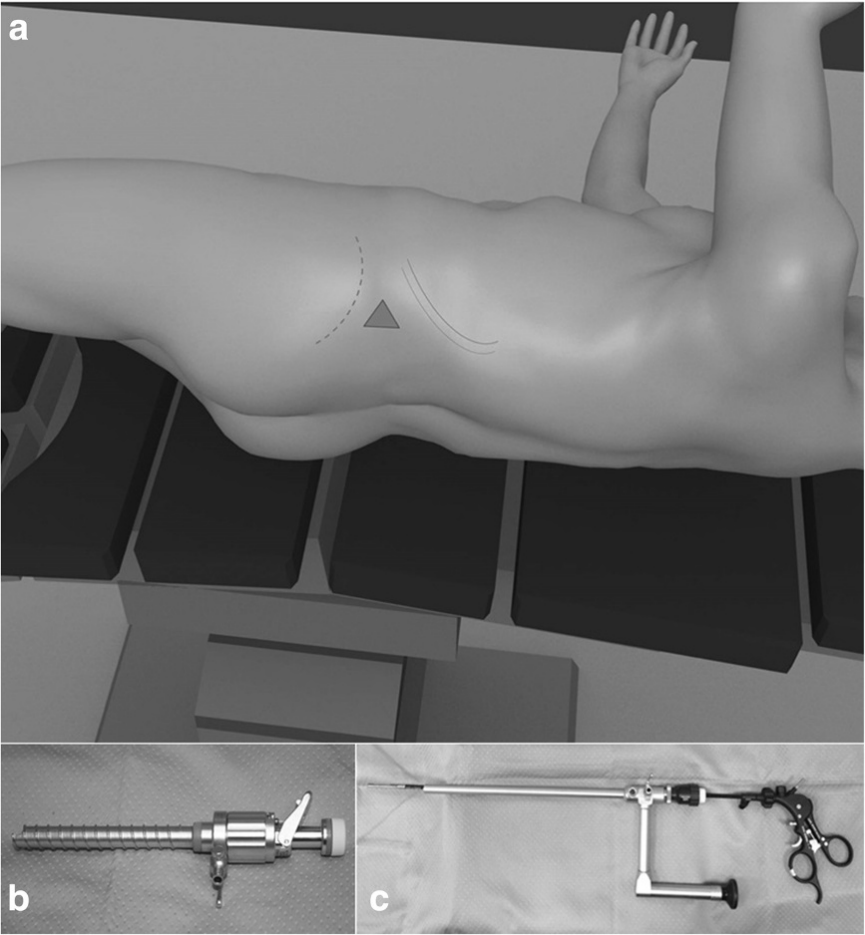
**Patient position and Surgical Instruments. a)** Petit’s triangle. **b)** Ternamian EndoTIP 10 mm, Karl Storz®, Tuttlingen, Germany. **c)** Operative laparoscope 10 mm, Karl Storz, Tuttlingen, Germany.

Our retroperitoneal access side was the lumbar or Petit’s triangle (Figure 1a), formed by the intersection of the external oblique and latissimus dorsi muscles at the iliac crest. With the patient in the standard flank position and the operating table flexed, the space between the 12th rib and iliac crest was maximized. This maneuver allowed the surgeon to identify Petit’s triangle [10].

In all cases retroperitoneal access was created with a laparoscopic visual reusable trocar, Ternamian EndoTIP 10 mm (Karl Storz®, Tuttlingen, Germany), and a 10 mm laparoscope was used for blunt dissection of the aereolar retroperitoneal fat (Figure 1b). Afterwards, a 10 mm operative laparoscope (Karl Storz®) with a 5 mm working channel was used to perform the first four procedures (Figure 1c). This single trocar technique was complex because light source was insufficient and visualization of the operating field was extremely poor. These two inconveniences resulted in prolonged operating times, and three cases necessitated addition of a 5 mm trocar.

**Figure 2:**
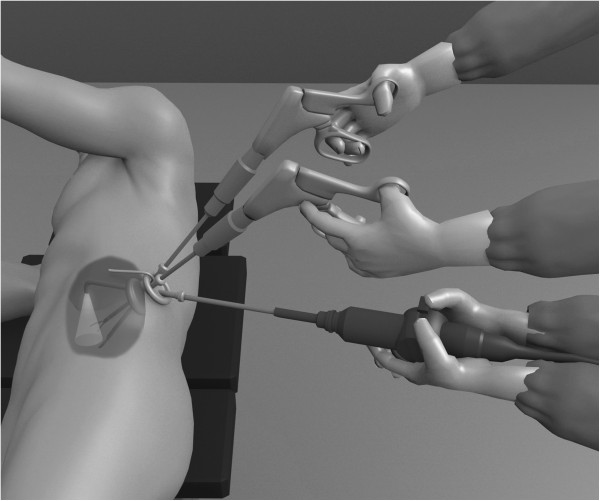
**Our working strategy with the EndoEye Camera (Olympus Medical, Orangeburg, NY, USA) was to place the lens in a different plane of the instruments and compensate with the flexible tip.**

In ten cases we used a single-trocar technique with a Multiport (Covidien SILS™ Port, Mansfield, MA, USA) placed in the Petit’s triangle. In three patients, a 5-mm flexible laparoscope EndoEye camera system (Olympus Medical®, Orangeburg, NY, USA) (Figure 2) was used, and in the remaining four subjects a bariatric 5 mm laparoscope (Karl Storz®) was used with standard, reusable, 5 mm laparoscopic instruments. In two patients 5 mm reusable and disposable bent instruments were used to dissect the retroperitoneal space and mobilize the lateral peritoneum from the anterior abdominal wall. In the remaining eight patients we used standard 5 mm laparoscopic instruments. A working pressure of 15 mmHg and the exposure of the lower pole of the kidney allowed us to perform the biopsy. The biopsy was done with 5 mm biopsy forceps: three bites placed in the renal cortex. We performed the hemostasis in the biopsy site with a 5 mm Argon beam probe and the application of oxidized regenerated cellulose gauze. The carbon dioxide was slowly evacuated while looking for any bleeding. Drainage was left in place for 12–24 hours in all patients. All procedures were performed by a single operator.

## Results

All 14 LESS biopsy procedures were successfully performed with sufficient tissue obtained for histopatological diagnosis in all cases (Table 1). The operative time ranged from 24 to 105 min (mean: 52.64 min), and the estimated blood loss was minimum. In the first four single-trocar cases, the procedure was complex because the light source was insufficient and visualization of the operating field was extremely poor. For this reason operative times was slightly longer and ranged from 75 to 105 min (mean: 88.75 min.). All patients were discharged following an overnight stay. None of the patients required narcotics or additional analgesia in the postoperative period. No postoperative complications occurred (Table 1).

## Discussion

Histological evaluation of renal parenchyma is often necessary in cases of several renal diseases. Pathological diagnosis often provides useful information in determining the prognosis and guiding the treatment [11]. General indications for renal biopsy include renal failure—insufficiency of unknown etiology, nephrotic syndrome, proteinuria, and systemic diseases with suspected renal involvement such as systemic lupus erythematosus. Percutaneous renal biopsy is the most common method of sampling renal tissue because it is performed with local anaesthesia as outpatient surgery [12].

In addition to the risk of bleeding and fistulae during percutaneous needle biopsy, the specimen can’t be adequate to have a histopathological diagnosis. In fact, during percutaneous procedures it is not easy obtain only cortex sample, that is necessary to study glomerular diseases. With LESS procedures, in our experience, we can select the best site in the kidney to perform the biopsy and we can take only cortex specimen, without medullary tissue.

In our experience percutaneous renal biopsy contraindications were uncontrolled hypertension, bleeding disorders, extreme obesity, and a solitary kidney.

With advances in endoscopic instrumentation and the development of laparoscopic techniques, the minimally invasive alternatives to open renal biopsy are safety and preferable. Although surgical approaches require general anesthesia, their advantage is that the kidney is identified, biopsied, and hemostasis is achieved under direct vision in a controlled fashion [4]. Several papers in the last 20 years appeared in the literature describing the retroperitoneoscopic approach as safe and effective. Retroperitoneal access it is technically difficult due to the lack of landmarks, small working space, and loss of orientation. Relative simple procedures like renal biopsy are often performed in a retroperitoneoscopic fashion. Data shows that this approach for renal biopsy is effective also in less experienced surgeons. With minimal retroperitoneal dissection, the kidney is quickly identified and renal biopsy and haemostasis are safely achieved in a reasonably short period of time [6–10].

As a result of the risks associated with additional ports, there has been a surge of interest in a less invasive alternative to retroperitoneoscopy. LESS has been developed in an attempt to further reduce the morbidity and scarring associated with laparoscopic surgery [13]. Early clinical series have demonstrated the feasibility of a broad range of LESS urologic procedures [14]. As a general principle, all eligible laparoscopic surgery patients can be considered for LESS depending on the surgeon’s experience.

In all patients the first trocar was positioned under direct vision using the Cannule Ternamian EndoTIP (Karl Storz®). This device allows the surgeon to open each tissue layer under direct vision, so that the surgeon has complete visual control to avoid blood vessels and nerves and to see the Scarpa’s fascia, the flank muscle, and the lumbodorsal fascia. Then, after insufflation with carbon dioxide at 15 mmHg, with this device we dissect bluntly the retroperitoneal space and mobilize the lateral peritoneal sheath from the anterior abdominal wall. This device is reusable, as compared to the Visiport access trocar (Covidien®) described in our previous experience in pediatric patients. This reusable device allows us to obtain results comparable to those with the Visiport access trocar, but with a reduction of the cost surgery [8].

Our preliminary approach with LESS technique was a single-trocar renal biopsy, performed with an operative laparoscope (Karl Storz®) with a 5 mm working channel. This attempt was not effective because the operative laparoscope brought insufficient light into the operative field and forced the operator to be positioned too close to the operative field, with the kidney causing lens mist and other residue. In these cases we performed a hybrid LESS, without problem for the patients. In fact, there were no reported any intraoperative complication in these four cases, except for a slightly prolonged operative time. In the remaining ten patients we used a SILS Multiport (Covidien SILS™ Port) placed in the Petit’s triangle. Today many LESS ports, disposable and reusable, are available in the market. In this series we used the SILS port because, in our experience, it seems to be easy to place, standard trocars (5–10 mm) can be used, and retro-pneumoperitoneum can be maintained without leakage.

Compared to standard laparoscopy, LESS technique increases the difficulty of surgical procedures because of reduced workspace, the lack of triangulation, clashing of instruments, as has been reported in several case series [8]. To reduce the risk of instruments clashing, we used two laparoscopes types: in four patients a bariatric 5 mm, 0° laparoscope (Karl Stortz®) was used, and in six patients a 5 mm flexible laparoscope EndoEye camera system (Olympus Medical) was used. With both lens devices we increase our working ability and improve our LESS technique. Moreover, the optic with which we obtained the best results was the EndoEye camera system. This device provides more light in the operating field, higher imaging quality, flexibility, and better working conditions. Our working strategy with the EndoEye system was to place the lens in a different plane of the instruments and compensate with the flexible tip (Figure 2).

Various disposable instruments have been developed to overcome the risk of clashing, minimal triangulation, and poor range of motion. Articulating instruments are designed to improve triangulation and external spacing for LESS procedures [15]. In one case we used the “Roticulator Endo Grasp” (Covidien®) forceps and scissor. We used them to mobilize the lower pole of the kidney, transect Gerota’s fascia, and develop a fat window on renal parenchyma. We experienced a poor quality of the materials with the consequent breakage and deformation of the instrument’s tip. In one other case we tested the pre-bent instruments (Olympus Medical). These instruments have been introduced with the aim of minimizing instrument clashing outside the port, and providing triangulation in the operative field and better force distribution during dissection with the transperitoneal approach [16]. In our experience these tools do not give better benefits because there is less working space in the retroperitoneal space compared to the abdominal cavity.

In our experience, all eligible laparoscopic surgery patients can be considered for LESS depending on the surgeon’s experience. According to recent updates from the Endourological Society NOTES (Natural Orifice Transluminal Endoscopic Surgery) and LESS Working Group and the European Society of Urotechnology NOTES, it has recently been stressed that LESS is appropriate in selected patients with limited previous abdominal surgery [17]. Greco et al. suggest that malignant disease at pathology and high ASA score represent predictive factors for complications after LESS for upper urinary tract surgery. Thus, surgeons approaching LESS should start with benign diseases in low-surgical risk patients to allow an easier surgical approach and to minimize the risk of postoperative complications [18].

## Conclusions

Retroperitoneoscopic LESS renal biopsy is a safe and feasible procedure. With consolidated laparoscopic experience with retroperitoneal access, LESS renal biopsy is a good choice of procedure. Moreover, all our subjects underwent same-day procedures, no pain medication were used after surgery, and subjects experienced excellent cosmetic results. Our next step will be a randomized prospective study of LESS compared with laparoscopy for renal biopsy.
